# *Bla*_PSZ-1_, a novel AmpC gene identified from a *Pantoea* isolate

**DOI:** 10.3389/fmicb.2023.1222703

**Published:** 2023-07-17

**Authors:** Jingxuan Zhao, Yuan Zhang, Yuning Sha, Naru Lin, Guozhi Zhang, Junwan Lu, Tingting Zhu, Xueya Zhang, Qiaoling Li, Hailin Zhang, Xi Lin, Kewei Li, Qiyu Bao, Li Lin

**Affiliations:** ^1^The Second Affiliated Hospital and Yuying Children’s Hospital, Wenzhou Medical University, Wenzhou, China; ^2^Key Laboratory of Medical Genetics of Zhejiang Province, Key Laboratory of Laboratory Medicine, Ministry of Education, Wenzhou, China; ^3^School of Laboratory Medicine and Life Sciences, Wenzhou Medical University, Wenzhou, China; ^4^Medical Molecular Biology Laboratory, School of Medicine, Jinhua Polytechnic, Jinhua, China

**Keywords:** *Pantoea*, resistance mechanism, **β**-lactamase gene, *bla*_PSZ-1_, kinetic parameter

## Abstract

**Background:**

*Pantoea* species of the family *Erwiniaceae* are well-known plant pathogens and animal and human conditional pathogens. Due to the widespread and continuous use of antimicrobials, multidrug-resistant strains continue to emerge, making clinical treatment difficult; therefore, there is an increasing need to clarify the mechanisms of drug resistance.

**Methods:**

A rabbit anal fecal sample was collected by a swab and the streak plate method was used to isolate single colonies. The standard agar dilution method was used to determine the minimum inhibitory concentrations (MICs) against antimicrobials. The complete genome sequence of the bacterium was obtained using Next-Generation Sequencing platforms. The potential resistance gene was annotated based on the Comprehensive Antibiotic Resistance Database (CARD) and verified by molecular cloning. The β-lactamase PSZ-1 was expressed via the pCold I expression vector and its enzyme kinetic parameters were analyzed. The genetic environment and evolutionary process of the novel resistance gene-related sequences were analyzed by bioinformatic methods.

**Results:**

The isolate *Pantoea endophytica* X85 showed some degree of resistance to penicillins as well as cephalosporins. A novel AmpC resistance gene, designated *bla*_PSZ-1_ in this research, was identified to be encoded in the plasmid (pPEX85) of *P. endophytica* X85. *Bla*_PSZ-1_ showed resistance to penicillins and several first-, second-and third-generation cephalosporins as well as aztreonam, but it did not show resistance to the fourth-generation cephalosporins or carbapenems tested. Enzyme kinetic assays revealed that it could hydrolyze amoxicillin, penicillin G, cephalothin, and cefazolin, and its hydrolytic activity could be strongly inhibited by the inhibitor avibactam, which was generally consistent with antimicrobial susceptibility testing results. No hydrolytic activity was observed for third-generation cephalosporins or aztreonam.

**Conclusion:**

In this study, a novel AmpC β-lactamase gene, designated *bla*_PSZ-1,_ was characterized and it was encoded in the plasmid of the bacterium *P. endophytica* X85. It shows resistance to penicillins and several cephalosporins. The discovery of novel drug resistance mechanisms can help guide the scientific use of drugs in animal husbandry and clinical practice, effectively avoiding the abuse of antimicrobials and thus preventing the further development and spread of bacterial resistance.

## Introduction

*Pantoea* species of the family *Erwiniaceae* are ubiquitous in the environment and are well-known plant pathogens (Walterson and Stavrinides, 2015). They have been isolated from contaminated soil, water, plants (as epiphytes or endophytes), seeds, dairy products, and from gastrointestinal tracts, blood, and urine of humans and animals (Fullerton et al., 2007; Dutkiewicz et al., 2016). They usually cause opportunistic infections, especially when the immune system is compromised; for example, they have been reported to cause blood infections in several newborn preterm infants (Habsah et al., 2005). The wider and even abused use of antimicrobials in clinical and agricultural farming caused the rapid emergence of bacterial resistance, and the emergence of multidrug resistance in human pathogens is considered to be a major public global health threat. Therefore, understanding the molecular mechanisms of bacterial drug resistance is crucial. Mechanisms of antimicrobial resistance can be mainly classified as follows: (i) modification of antibiotic molecules to inactivate them, such as the production of β-lactamases and aminoglycoside-modifying enzymes, (ii) reduction of antibiotic penetration (reduction of pore protein-mediated outer membrane permeability) and efflux mechanisms, and (iii) alteration of target sites (Munita and Arias, 2016). Among them, the presence of β-lactamases is a very common mechanism of drug resistance in pathogenic bacteria. β-lactamase is an enzyme that inactivates β-lactam antibiotics by hydrolyzing the β-lactam ring (Bush, 2018) and is classified by Ambler into class A to class D according to amino acid sequence homology (conserved and distinguished amino acid motifs; Bush and Jacoby, 2010). AmpC β-lactamases are commonly produced by many *Enterobacteriaceae* and a few other organisms and are encoded by *bla* genes located on bacterial chromosomes and, to a lesser extent, by plasmids. Organisms expressing AmpC enzymes are usually resistant to penicillins, β-lactamase inhibitors (clavulanic acid and tazobactam), and most cephalosporins, including cefoxitin, ceftriaxone, and cefotaxime (Bush et al., 1995). In addition, AmpC β-lactamases have a poor ability to hydrolyze the extended-spectrum cephalosporin cefepime and are readily inactivated by carbapenems. Notably, AmpC β-lactamases are strongly inactivated by avibactam (Jacoby, 2009).

Ampicillin, amoxicillin, and first-generation cephalosporins such as cefazolin and cephalothin are very strong inducers of AmpC enzymes and good substrates for hydrolysis. Cefoxitin and imipenem are also strong inducers but are more stable for hydrolysis. Cefotaxime, ceftriaxone, ceftazidime, cefepime, cefuroxime, piperacillin, and aztreonam are weak inducers and substrates but can also be hydrolyzed if sufficient enzyme expression is present. Thus, the minimum inhibitory concentrations (MICs) of weakly induced oxyimino β-lactams increase dramatically in the presence of AmpC overproduction (Jacoby, 2009).

In the present study, we described a novel AmpC enzyme gene, designated *bla*_PSZ-1,_ which was harbored on a strain of the genus *Pantoea* isolated from a rabbit in a livestock farm in Wenzhou, China. Its enzymatic kinetic parameters were also determined. The identification of this novel resistance gene from an animal bacterium is of great value for our in-depth understanding of bacterial drug resistance and its dissemination pattern, as well as for the clinical treatment of infectious diseases caused by the related bacteria.

## Materials and methods

### Bacteria and plasmids

*P. endophytica* X85 carrying the novel drug-resistance gene *bla*_PSZ-1_ was isolated from the fecal swab of a rabbit at a livestock farm during a survey on the antimicrobial resistance of bacteria in Wenzhou, southern China. The genomes of the isolates were sequenced and their resistance profiles were determined. The relationship between the resistance genotypes and phenotypes was further analyzed. Species identification of the isolate was performed first by 16S rRNA gene homology and then by genome-wide average nucleotide identity (ANI) analyses. The bacterial strains and plasmids used in the study are listed in Table 1.

**Table 1:** Bacterial strains and plasmids used in this study.

Strains and plasmids	Description	Source
X85	The wild-type strain of *P. endophytica* X85	Isolated from a rabbit anal fecal swab
DH5α	*Escherichia coli* DH5α as a host cell for cloning of the *bla*_PSZ-1_ gene	Our laboratory collection
BL21	*Escherichia coli* BL21 as a host cell for expression of PSZ-1	Our laboratory collection
ATCC 25922	*Escherichia coli* ATCC 25922 as quality control for antimicrobial susceptibility testing	Our laboratory collection
pUCP24-*bla*_PSZ-1_/DH5α	The DH5α cell carrying the recombinant plasmid pUCP24-*bla*_PSZ-1_	Constructed in this research
pCold I-blaPSZ_−1_/BL21	The BL21 cell carrying the recombinant plasmid pCold I-*bla*_PSZ-1_	Constructed in this research
pUCP24	Cloning vector for the PCR product of the *bla*_PSZ-1_ gene with its upstream promoter region, GEN^r^	Our laboratory collection
pCold I	Expression vector for the PCR product of the ORF of the *bla*_PSZ-1_ gene, AMP^r^	Our laboratory collection

r, resistance; GEN, gentamicin; AMP, ampicillin; ORF, open reading frame.

### Antimicrobial susceptibility testing

Following the Clinical and Laboratory Standards Institute (CLSI) guidelines, MICs were determined on Mueller-Hinton (M-H) agar using the standard agar dilution method, and susceptibility patterns were explained in accordance with CLSI M100 (31st Edition, 2021). The reference strain used for quality control in this study was *E. coli* ATCC 25922. The antimicrobials tested in this research included 13 β-lactams, 4 aminoglycosides, and 2 β-lactamase inhibitors (Table 2). All antibiotics were for human clinical use and purchased from pharmacies and hospitals, and MIC values were the mean values of three independent measures.

**Table 2:** Minimum inhibitory concentrations of antimicrobials for *P. endophytica* X85, the recombinant carrying *bla*_PSZ-1_, and the control strains (μg/mL).

Antibiotic	*P. endophytica* X85	pUCP24-*bla*_PSZ-1_ /DH5α	pUCP24/DH5α	DH5α	ATCC25922
Ampicillin	64	128	4	4	4
Penicillin G	256	1,024	8	16	16
Amoxicillin	>2,048	512	2	4	8
Piperacillin	16	32	4	4	4
Cefazolin	32	32	1	1	2
Cephalothin	32	512	<2	<2	8
Cefoxitin	64	16	2	2	2
Ceftazidime	1	4	0.06	0.06	0.125
Cefotaxime	0.25	1	0.06	0.06	0.06
Ceftriaxone	0.25	1	0.03	0.03	0.06
Cefepime	0.03	0.03	0.016	0.016	0.03
Aztreonam	0.125	4	0.06	0.06	0.125
Meropenem	0.06	0.015	0.015	0.015	0.015
Piperacillin-Tazobactam	2	16	<1	2	4
Aztreonam-Avibactam	0.06	0.03	<0.015	0.03	0.06
Streptomycin	8	–	–	16	16
Tobramycin	0.125	–	–	0.5	0.5
Amikacin	0.5	–	–	1	1
Kanamycin	<0.25	–	–	1	2

### Cloning and expression of the *bla*_PSZ-1_ gene and purification of PSZ-1

The *bla*_PSZ-1_ gene and its upstream promoter region were amplified using 2× Phanta^®^ Max Master Mix (Nanjing Vazyme Biotech Co., Ltd., Nanjing, China), and *P. endophytica* X85 genomic DNA was extracted using the Generay Genomic DNA Miniprep kit (Shanghai Generay Biotech Co., Ltd., Shanghai, China) and used as the template for PCR. The primers with restriction endonuclease adaptors at the 5′-end (*Xba*I for the forward primer and *Hind*III for the reverse primer) are listed in Table 3. PCR products were digested with *Xba*I and *Hind*III and linked to the cloning vector pUCP24, which was also digested with *Xba*I and *Hind*III using a T4 DNA ligase cloning kit (Takara Bio, Inc., Dalian, China). The recombinant plasmid was transformed into *E. coli* DH5α by the calcium chloride method and the transformants were then screened on Luria-Bertani (LB) agar plates supplemented with gentamicin (40 μg/mL). Individual colonies were inoculated into LB mediums supplemented with the same antibiotic and cultured overnight, and then the inserts were verified by PCR and Sanger sequencing (Shanghai Sunny Biotechnology Co., Ltd., Shanghai, China).

**Table 3:** Primers for cloning the *bla*_PSZ-1_ gene.

Primers^a^	Sequence (5′ → 3′)	Restriction endonuclease adaptor	Vector	Annealing temperature (°C)	Amplicon size (bp)
pr-*bla*_PSZ-1_-*Xba*I-F	TGCTCTAGATGCAGCTCAATCGCCGCATTAGTAAAACTT	*Xba*I	pUCP24	68	1,443
pr-*bla*_PSZ-1_-*Hind*III-R	CCCAAGCTTTTCACGTCGCGTTTATCTTTAGGGTGATTG	*Hind*III	pUCP24	68	1,443
orf-*bla*_PSZ-1_-*Kpn*I-F	CGGGGTACCCTGGTGCCGCGCGGCAGCATGGCGTTTGCCACCACGGCAGAT	*Kpn*I + Thrombin	pCold I	66	1,086
orf-*bla*_PSZ-1_-*Hind*III-R	CCCAAGCTTTTACTGCAACGCTTTCAGAATCTGCATCGC	*Hind*III	pCold I	66	1,086

a Primers starting with “pr” were used to clone the bla_PSZ-1_ gene and its upstream promoter region; primers starting with “orf” were used to clone the ORF of the bla_PSZ-1_ gene.

The corresponding primers (Table 3) were used to amplify the ORF of *bla*_PSZ-1_ containing the thrombin digestion site by PCR, which was inserted into the pCold I expression vector, and the recombinant plasmid (pCold I-*bla*_PSZ-1_) was transformed into *E. coli* BL21 competent cells. For the expression of the PSZ-1 protein, *E. coli* BL21 cells with verified recombinant plasmids were cultured overnight in LB mediums supplemented with ampicillin (100 μg/mL), diluted 100 times in fresh mediums, and incubated at 37°C. After incubation in an ice bath for more than 30 min, the OD_600_ of the culture reached 0.6 to 0.8 and protein expression was induced by the addition of 1 mM isopropyl-β-dithiogalactopyranoside (IPTG, Sigma Chemicals Co., St. Louis, MO, United States), and further incubated for approximately 20 h at 15–16°C. Cells were collected by centrifugation (5,000 × g, 10 min) at 4°C, resuspended in lysis buffer (20 mM Tris–HCl, 150 mM NaCl, 3 mM β-mercaptoethanol, 0.5% Nonidet-P-40; pH 8.0), and lysed by ultrasound. After the cell fragments were removed by centrifugation (12,000 × g, 30 min) at 4°C, the lysates were combined with pre-balanced nickel-nitrilotriacetic acid (Ni-NTA) agarose resin (Beyotime Biotechnology, Shanghai, China) at 4°C and shaken gently overnight. Then, the recombinant protein was purified by standard Ni-NTA affinity chromatography and digested with thrombin (Solarbio Science & Technology Co., Ltd., Beijing, China) at 37°C for 24 h to remove the His-tag. The purity of PSZ-1 protein was validated by SDS–PAGE with a 12% acrylamide separation gel and Coomassie blue G-250 staining, and the protein concentration was determined by a BCA protein assay kit (Thermo Fisher Scientific, Rockford, IL, United States).

### Enzyme kinetic parameter analysis

Kinetic parameters of the purified β-lactamase PSZ-1 against β-lactam antimicrobials were determined at 37°C, in a 10 mM phosphate buffer (pH 7.4), and a final reaction volume of 200 μL on a Synergy™ Neo2 Multi-Mode Microplate Reader (BioTek Instruments, Inc., United States). The steady-state kinetic parameters *k*_cat_ and *K*_m_ were determined by non-linear regression of the initial reaction rates with the Michaelis–Menten equation in Prism (version 8.0.2) software (GraphPad Software, CA, United States).

The concentrations of the β-lactamase inhibitors avibactam and tazobactam, leading to a 50% reduction in the hydrolysis of nitrocefin (IC_50_), were measured after 5 min of preincubation of the enzymes with the inhibitors at 37°C, and nitrocefin (0.1 mM) was used as the substrate. The IC_50_ values were determined by non-linear regression analysis (GraphPad Prism, version 8.0.2) using log (inhibitor) vs. response (three parameters). Values are the average of three independent measures.

### Whole-genome sequencing and sequence analysis

The total bacterial DNA of *P. endophytica* X85 was extracted from an individual colony subcultured in LB at 37°C for approximately 16 h by using the Generay Genomic DNA Miniprep kit (Shanghai Generay Biotech Co., Ltd., Shanghai, China). Genomic DNA was sequenced by both the Illumina HiSeq 2,500 and PacBio RS II platforms, with a read length of PE150 and 10 kb, respectively, and a sequencing depth of 150× for both (Shanghai Personal Biotechnology Co., Ltd., China). Unicycler v0.4.8 was used to hybrid assemble the PacBio long reads and confirm the cyclization of the whole-genome assembly (Wick et al., 2017). Pilon improves the quality of genomic assembly sketches by mapping Illumina short reads onto the assembly to correct possible incorrect assembly (Walker et al., 2014). Genes were predicted and annotated by using Prokka v1.14.6 (Seemann, 2014); furthermore, DIAMOND v2.0.11 was used to search the predicted proteins in the NCBI non-redundant protein databases with an e-value threshold of 1e-5 (Buchfink et al., 2021). The 16S rRNA homology analysis was performed by comparing the 16S rRNA sequences extracted from the Prokka-annotated genome of *P. endophytica* X85 with those in the 16S ribosomal RNA sequence database in NCBI. Drug resistance gene identifier v5.2.02 and CARD were used to annotate drug resistance genes (McArthur et al., 2013). FastANI was used to calculate the ANI (Jain et al., 2018) and dDDH was calculated through the Type Strain Genome Server (TYGS) online database^1^ (Meier-Kolthoff et al., 2022). Multiple sequence alignments of PSZ-1 and its relatives from the Beta-Lactamase Database^2^ and UniProt/Swiss-Prot database^3^ were performed by MAFFT v7.490 (Katoh and Standley, 2013) and then a msa R package was used to visualize the comparison results and embellish the generated visual figure (Bodenhofer et al., 2015). MEGA11 was used to construct a neighbor-joining (N-J) phylogenetic tree including PSZ-1 and other functionally characterized β-lactamases (Kumar et al., 2018). The generated tree was visualized using the online tool iTol^4^ (Letunic and Bork, 2007). The figure depicting the genetic environments around the *bla*_PSZ-1_ and *bla*_PSZ-1_-like genes was generated by clinker v0.0.24 (Gilchrist and Chooi, 2021). GView was used to construct the basic genomic characteristics and comparative genomes of *P. endophytica* X85 (Stothard et al., 2019). ProtParam^5^ was used to predict the molecular weight and pI value of PSZ-1 (Wilkins et al., 1999).

### Accession numbers

The complete chromosome, plasmid, and *bla*_PSZ-1_ gene sequences of *P. endophytica* X85 have been submitted to GenBank, and the accession numbers are CP121108, CP121109, and OQ725878, respectively.

## Results and discussion

### Identification and genome characterization of the isolate *Pantoea endophytica* X85

The 16S rRNA gene homology analysis revealed that the 16S rRNA gene of the strain X85 had the highest homology with that of *Pantoea endophytica* 596 T (NR_178843.1); the coverage was 100.00%, and the identity was 100% (Supplementary Table S3). Furthermore, the ANI analysis between the genomes of *P. endophytica* X85 and all 863 genomes from *Pantoea* downloaded from the NCBI databases demonstrated that among them, 14 genomes with ≥95% ANI (the cutoff to define a classified bacterial species) were found (Jain et al., 2018), of which 3 were from the *Pantoea endophytica* species (*Pantoea endophytica* 596 T, *Pantoea endophytica* HN-23, and *Pantoea endophytica* RIT-836 with ANIs of 98.29, 98.28 and 95.68%, respectively), and the remaining 11 genomes were all species-undetermined ones of genus *Pantoea* (Supplementary Table S1). The digital DNA–DNA hybridization (dDDH) analysis results obtained by TYGS showed that *P. endophytica* X85 shared the highest identity (86.00%) with *Pantoea endophytica* 596 T (GCA_002858935.1), which was higher than the cutoff (70%) to classify a bacterial species (Goris et al., 2007; Supplementary Table S4). Therefore, based on the results mentioned above, the isolate X85 was finally designated *P. endophytica* X85.

The complete genome of *P. endophytica* X85 consisted of a chromosome and a plasmid named pPEX85. The size of the chromosome was approximately 4.22 Mb and encoded approximately 4,954 ORFs with an average GC content of 55.06%. The length of the plasmid was 771,939 bp, encoding approximately 720 ORFs, of which 540 (75.0%) were predicted to encode function-known proteins (Table 4).

**Table 4:** General features of the *P. endophytica* X85 genome.

	Chromosome	pPEX85
Size (bp)	4,217,669	771,939
GC content (%)	55.06	54.28
Predicted coding sequences (CDSs)	3,954	720
Known proteins	3,392 (85.79%)	540 (75.00%)
Hypothetical proteins	562 (14.21%)	180 (25.00%)
Protein coding (%)	96.32	99.72
Average ORF length (bp)	933.10	952.20
Average protein length (aa)	314.20	317.10
tRNAs	78	0
rRNA operons	(16S-23S-5S) *6 (16S-23S-5S-5S) *1	0

### The resistance profile of *Pantoea endophytica* X85

Of the 17 antimicrobials tested, including 13 β-lactams and 4 aminoglycosides, the wild-type *P. endophytica* X85 exhibited the highest MIC level for amoxicillin (> 2048 μg/mL) and higher MIC levels for penicillin G (256 μg/mL), cefoxitin (64 μg/mL), ampicillin (64 μg/mL), cefazolin (32 μg/mL), and cephalothin (32 μg/mL) (Table 2). When analyzing the relationship between the drug resistance phenotype and genotype, especially for β-lactam antibiotics, we found that there was no functionally characterized β-lactam resistance gene annotated from the whole genome sequence. However, it had six predicted β-lactamase genes annotated in the genome, of which one gene showed the highest amino acid similarity of 75.13% (a coverage of 93.0% and an identity of 80.79%) with the function-characterized β-lactamase gene *bla*_ERH-1_, which is described to confer resistance to some penicillin and cephalosporin antibiotics (Naas et al., 2004; Supplementary Table S2). The *bla*_ERH-1_ homologous gene of this research was finally designated *bla*_PSZ-1_.

### Resistance function characterization of the novel β-lactam resistance gene *bla*_PSZ-1_

To verify the resistance function of the gene, we cloned the ORF of *bla*_PSZ-1_ and its promoter region into the clone vector pUCP24, and the recombinant plasmid was transformed into *E. coli* DH5α competent cells. The transformant (pUCP24-*bla*_PSZ-1_/DH5α) conferred resistance to ampicillin, penicillin G, amoxicillin, cefazolin, cephalothin, cefoxitin, ceftazidime, cefotaxime, ceftriaxone, and aztreonam but not meropenem (Table 2). Compared with the control strain (pUCP24/DH5α), the MIC levels of the recombinant harboring *bla*_PSZ-1_ increased more than 8-fold for most β-lactam antibiotics, especially cephalothin (>256-fold), amoxicillin (≥256-fold), penicillin G (128-fold), ceftazidime (64-fold), aztreonam (64-fold), ampicillin (32-fold), cefazolin (32-fold), and ceftriaxone (32-fold) (Table 2). However, the MIC level of carbapenem meropenem was not different for the recombinant strain compared to the control strain. Tazobactam, a classical class A β-lactamase inhibitor, had a poor inhibitory effect on the resistance activity of *bla*_PSZ-1_, whereas the MIC level of *bla*_PSZ-1_ against aztreonam was significantly reduced in the presence of avibactam. When comparing the antimicrobial resistance spectrum of *bla*_ERH-1_ (ERH-1 shared the highest amino acid sequence similarity with PSZ-1) and *bla*_PSZ-1_, *bla*_ERH-1_ did not show resistance to the second-generation cephalosporin cefoxitin, whereas *bla*_PSZ-1_ did. However, *bla*_ERH-1_ demonstrated resistance to carbapenems (Naas et al., 2004) but *bla*_PSZ-1_ did not.

### Kinetic parameters of PSZ-1

The length of the *bla*_PSZ-1_ gene was 1,086 bp, and it encoded an AmpC β-lactamase of 361 amino acids. The predicted molecular weight of mature β-lactamase PSZ-1 was 39.68 kDa, and the pI was 9.14. The kinetic parameters of the purified PSZ-1 demonstrated different degrees of hydrolytic activities against penicillins and narrow-spectrum cephalosporins. Among them, the strongest hydrolytic activity (*k*_cat_*/K*_m_ 1,088.21 ± 140.96 mM^−1^·s^−1^) was observed for cephalothin. PSZ-1 showed moderate hydrolytic activities for penicillin G (*k*_cat_*/K*_m_ 176.08 ± 4.90 mM^−1^·s^−1^) and the first-generation cephalosporin cefazolin (*k*_cat_*/K*_m_ 123.82 ± 5.75 mM^−1^·s^−1^). However, PSZ-1 showed almost no hydrolytic activity against the third-generation cephalosporin cefotaxime or monoamide ring beta-lactam class antibiotic aztreonam (Table 5). Notably, the kinetic hydrolytic activity results of the enzyme were not completely consistent with the MIC levels of the recombinant (pUCP24-*bla*_PSZ-1_/DH5α) compared with the control strain. The hydrolytic activity of the PSZ-1 β-lactamase for cefotaxime or aztreonam was not detectable, which contrasted with the increased MIC levels (increased MIC levels of 16-and 64-fold, respectively) of the recombinant with cloned *bla*_PSZ-1_ to the two antimicrobials. This discrepancy between *in vivo* and *in vitro* results might be attributed to the fact that, despite several attempts, for weak substrates (e.g., cefotaxime or aztreonam), β-lactamase production may be excessively induced *in vivo* (even up to 260-fold), whereas *in vitro* experiments were unable to enrich a sufficient amount of β-lactamase production for us to observe the detectable hydrolysis of the enzyme against the antibiotics (Lakaye et al., 1999). A similar phenomenon was reported in a study on the AmpC enzyme CDA-1 (Ammenouche et al., 2014). When comparing the hydrolytic activity of PSZ-1 with CDA-1, which is another AmpC β-lactamase that shares an amino acid similarity of 65.65% with PSZ-1, the highest among the β-lactamases that had the kinetic parameters characterized, PSZ-1 showed lower hydrolytic activity than CDA-1 toward cefoxitin (*k*_cat_*/K*_m_ of 19.21 vs. 840 mM^−1^·s^−1^) and cephalothin (*k*_cat_*/K*_m_ of 1,088.21 vs. 14,500 mM^−1^·s^−1^). In accordance with the kinetic parameters, the MIC result of the recombinant strain with the cloned *bla*_PSZ-1_ against cefoxitin was also significantly lower than that of *bla*_CDA-1_ (increased 8-fold vs. >256-fold). However, the MIC values of *bla*_PSZ-1_ and *bla*_CDA-1_ to cephalothin did not seem to vary much, and both of them increased >256-fold to cephalothin compared to the controls.

**Table 5:** Kinetic parameters of PSZ-1 for β-lactam antibiotics.

Substrate	*k*_cat_ (s^−1^) ^a^	*K*_m_ (μM^−1^) ^a^	*k*_cat_*/K*_m_ (mM^−1^·s^−1^) ^a^
Cefazolin	23.34 ± 1.74	132.90 ± 13.29	176.08 ± 4.90
Cefoxitin	0.09 ± 0.01	4.82 ± 1.25	19.21 ± 2.55
Cephalothin	26.14 ± 1.30	24.53 ± 4.00	1088.21 ± 140.96
Amoxicillin	2.89 ± 0.04	171.10 ± 7.30	16.91 ± 0.51
Penicillin G	11.04 ± 0.77	89.67 ± 10.54	23.82 ± 5.75
Cefotaxime	NH ^b^	NH ^b^	NH ^b^
Aztreonam	NH ^b^	NH ^b^	NH ^b^

a Values are means ± standard deviations.

b NH, no detectable hydrolysis.

When analyzing the inhibitory effect of the β-lactamase inhibitors on PSZ-1, avibactam had a strong inhibitory effect on PSZ-1 (IC_50_: 0.0555 μM), whereas tazobactam had a weaker inhibitory effect (IC_50_: 7.96 μM) when the concentration of PSZ-1 was 0.00214 μM and the nitrocefin substrate concentration was 100 μM. This result is consistent with the properties of AmpC β-lactamases for inhibitors.

### Evolution and structure analysis of PSZ-1 and *bla*_PSZ-1_ related sequences

**Figure 1:**
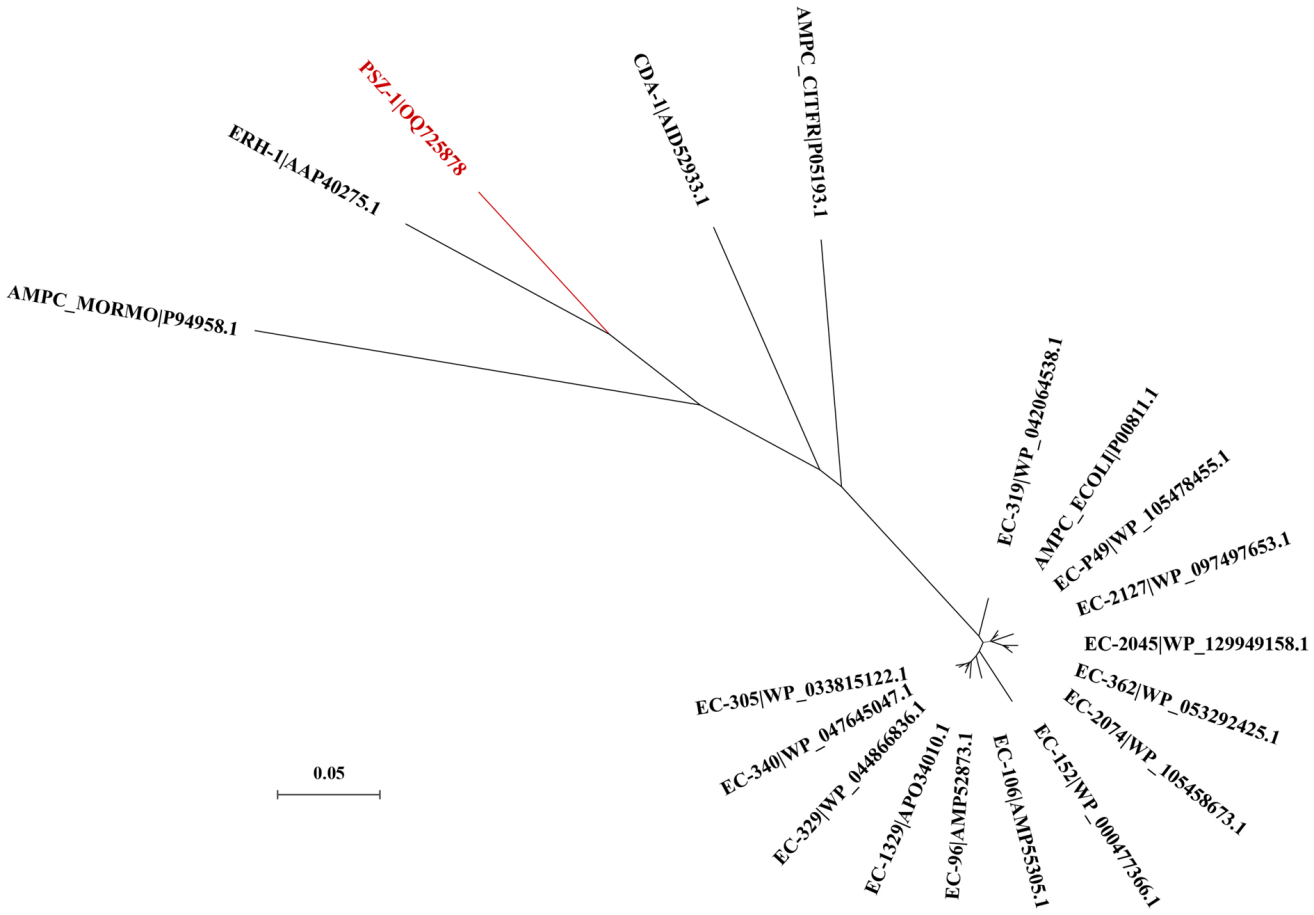
A phylogenetic tree showing the relationship of PSZ-1 (OQ725878) with other functionally characterized β-lactamases. PSZ-1 is highlighted in red. Other β-lactamases include ERH-1 (AAP40275.1), AMPC_ECOLI (P00811.1), EC-P49 (WP_105478455.1), EC-152 (WP_000477366.1), AMPC_MORMO (P94958.1), EC-2045 (WP_129949158.1), EC-96 (AMP52873.1), EC-329 (WP_044866836.1), EC-106 (AMP55305.1), AMPC_CITFR (P05193.1), and CDA-1 (AID52933.1).

**Figure 2:**
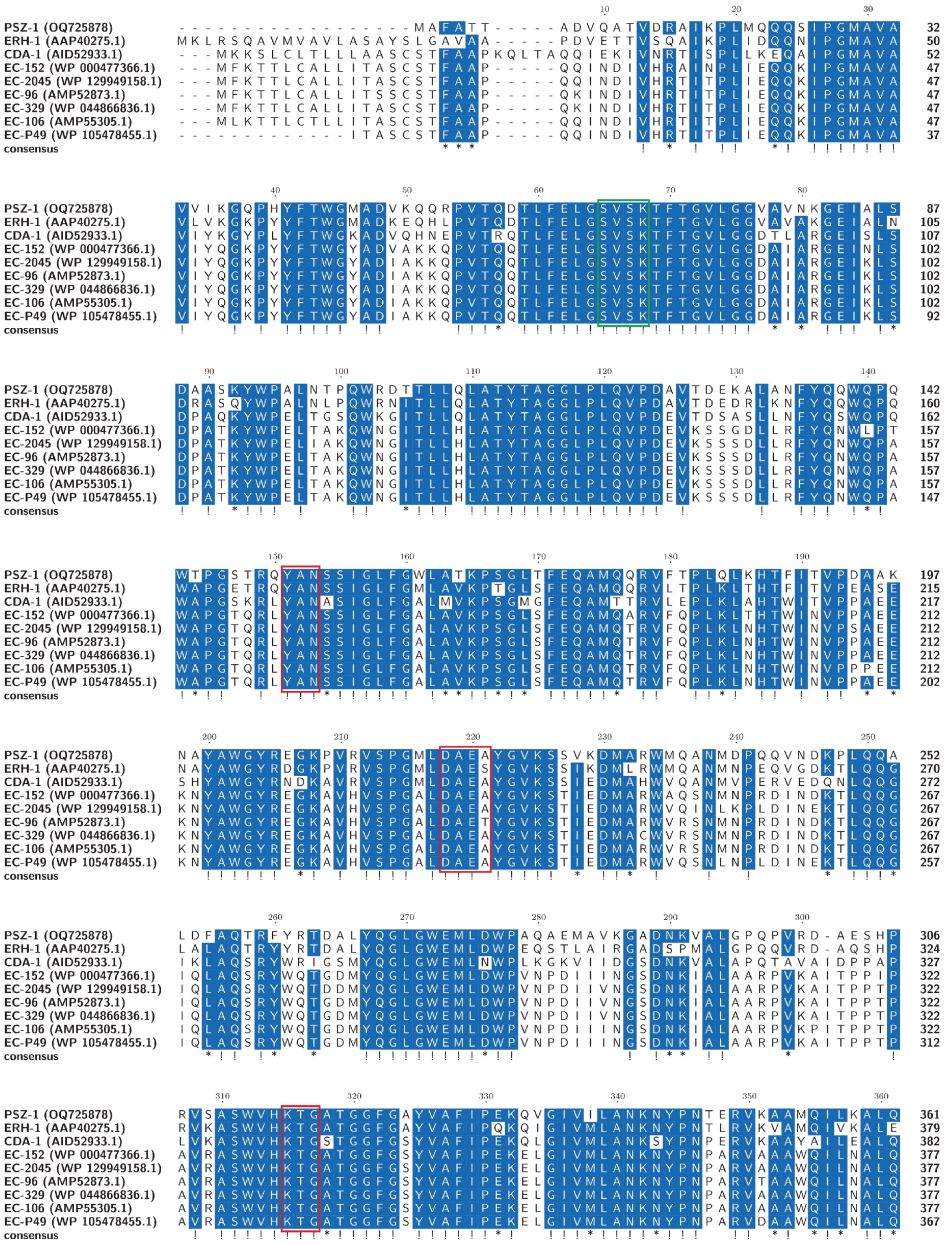
Multiple alignments of the deduced amino acid sequences of PSZ-1 and its close relatives. Exclamations indicate fully conserved residues, asterisks indicate strong similar residues, and gaps are represented using hyphens. The numbers on the right represent the corresponding sequence length. Three conserved motifs of class C β-lactamases are boxed in red. The β-lactamase characteristic serine active site is boxed in green.

The evolutionary relationship analysis revealed that PSZ-1 formed a new branch in the phylogenetic tree of the function-characterized AmpC β-lactamases (Figure 1). Sequence comparison analysis between PSZ-1 and these function-characterized β-lactamases in the Beta-Lactamase DataBase and UniProt/Swiss-Prot database together revealed that PSZ-1 shared higher amino acid sequence similarities with ERH-1 (75.13%), CDA-1 (65.65%), EC-152 (65.65%), EC-2045 (65.37%), EC-96 (65.10%), EC-329 (65.10%), EC-106 (64.82%), and EC-P49 (64.82%; Figure 2). Within the deduced amino acid sequence of the protein, a serine-valine-serine-lysine tetrad (S-V-S-K) with the conserved and characteristic serine and lysine amino acid residues of β-lactamases possessing a serine active site was found at positions 65 to 68. Additionally, three motifs characteristic of class C β-lactamases (cephalosporinases) were also found: YAN (tryptophan-alanine-asparagine) at positions 151 to 153, DAEX (aspartic acid-alanine-glutamic acid-xaa) at positions 218 to 221, and KTG (lysine-threonine-glycine) at positions 315 to 317 (Joris et al., 1988; Mack et al., 2020; Figure 2).

**Figure 3:**
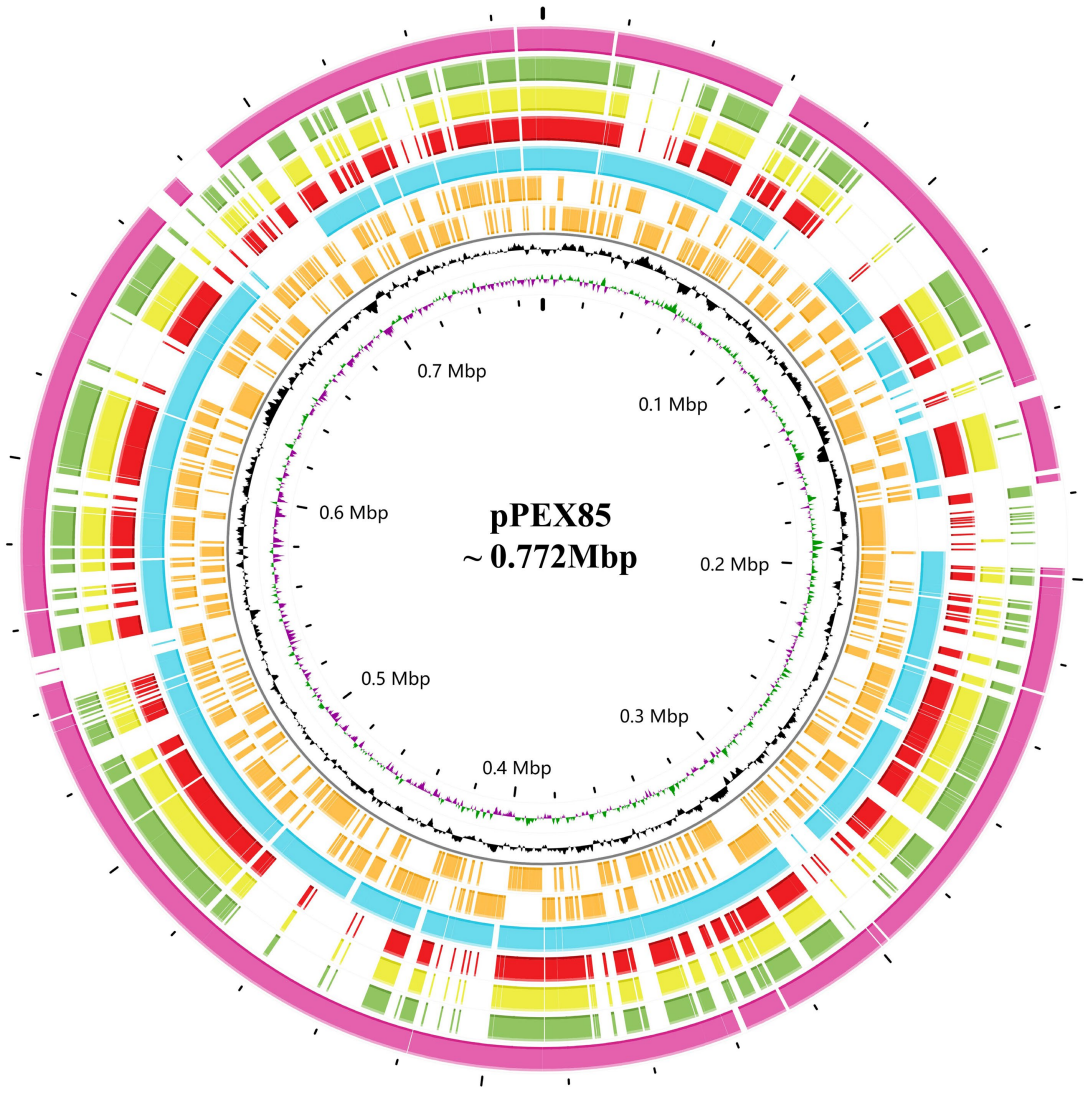
Genomic comparison of *P. endophytica* X85 plasmid with its close relatives. From outside to inside. Circles 1 to 5 are homologous regions of *Pantoea* sp. SOD02 plasmid (CP102605), *P. dispersa* YSD_J2 plasmid (CP074351.1), *Pantoea dispersa* Lsch plasmid (CP082347.1), unnamed *P. dispersa* AHKW2b plasmid (CP082342.1), and *Pantoea* sp. SO10 plasmid (NZ_CP040096). They are compared to *P. endophytica* X85 plasmid with unmatched regions left blank. Circles 6 and 7 are the genes encoded in the forward and reverse strands, respectively, circles 8 and 9 represent the GC content and GC skew, respectively, and circle 10 shows the scale in Mb.

To analyze the structure of the *bla*_PSZ-1_-related sequences, comparative analyses of the *bla*_PSZ-1_-encoding plasmid and the gene context were carried out. We found that five sequences sharing ≥45.0% similarities with *P. endophytica* X85 plasmid (CP121109) were present in the NCBI nucleotide database, all of which were complete plasmids from the genus *Pantoea.* Among them, the plasmid from *Pantoea* sp. SOD02 (CP102605, 926,844 bp in length approximately 180 kb larger than pPEX85) shared significantly higher similarities, of 62.20%, with pPEX85, while the other four, namely, the plasmid from *Pantoea dispersa* YSD_J2 (CP074351.1, 710,238 bp in length nearly the same size as pPEX85), the plasmid from *P. dispersa* Lsch (CP082347.1, 689,940 bp in length approximately 80 kb smaller than pPEX85), the unnamed plasmid from *P. dispersa* AHKW2b (CP082342.1, 653,898 bp in length approximately 120 kb smaller than pPEX85), and the plasmid from *Pantoea* sp. SO10 (NZ_CP040096, 744,154 bp in length, nearly the same size as pPEX85), shared similar sequence similarities of, respectively, 49.80, 49.70, 49.50, and 48.30% with pPEX85 (Figure 3). These sequences did not contain the *bla*_PSZ-1_ gene, except for *Pantoea* sp. SO10 and *Pantoea* sp. SOD02.

To analyze the genetic environment of *bla*_PSZ-1_, we first searched the NCBI nucleotide database with the *bla*_PSZ-1_ gene as a query and collected sequences carrying a *bla*_PSZ-1_-like gene that shared a nucleotide sequence similarity higher than 75.0% with *bla*_PSZ-1_. Among them, only the sequences longer than 20 kb with a *bla*_PSZ-1_-like gene at the center were kept, and finally, a total of four sequences were left for further analysis. When the five 20 kb sequences (including one of the present study) were analyzed, it was found that two of them shared >75.0% nucleotide sequence similarities with the 20 kb sequence of *P. endophytica* X85, whereas the similarities between the sequence of *P. endophytica* X85 and any of the remaining two were less than 50.0%.

Among the five sequences, three (including one of this study) were from the same genus *Pantoea,* and only one of the three, namely, the one in the present study, has been classified as a definite species. The 20 kb sequence from *Pantoea* sp. SO10 plasmid (NZ_CP040096) was particularly similar (99% coverage and 97.94% identity) to that of the present study, while the 20 kb sequence from *Pantoea* sp. SOD02 plasmid (CP102605) showed a lower similarity (88% coverage and 87.30% identity) with the sequence in this study. The ANI between *P. endophytica* X85 and *Pantoea* sp. SO10 (GCA_005281435.1) was 98.29%, and the two might belong to the same species, whereas the ANI between *P. endophytica* X85 and *Pantoea* sp. SOD02 (GCA_024707505.1), or between *Pantoea* sp. SO10 (GCA_005281435.1) and *Pantoea* sp. SOD02 (GCA_024707505.1) was less than 95.0 (93.49 and 93.51%, respectively). This result indicated that *Pantoea* sp. SOD02 was not of the same species as either of the other two.

**Figure 4:**
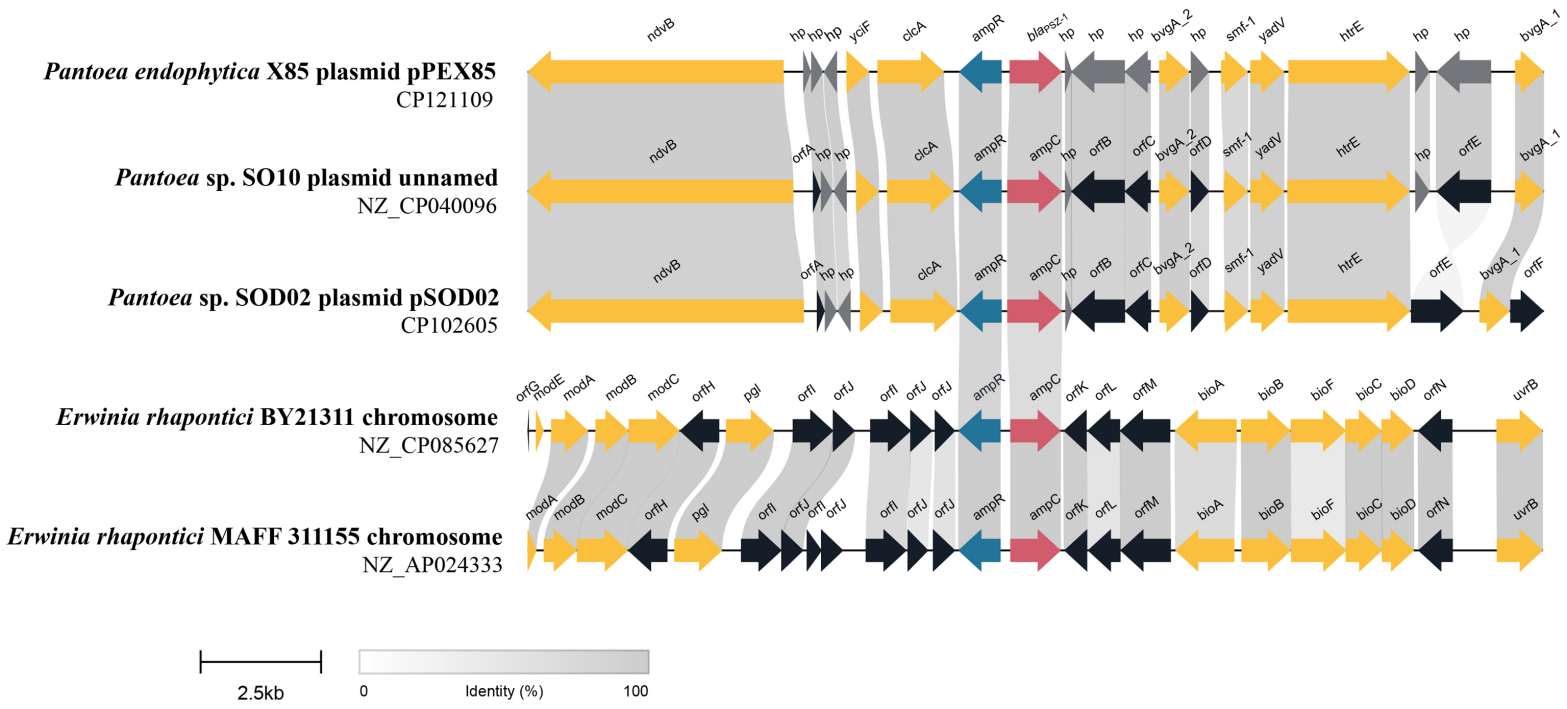
Genetic environment of the *bla*_PSZ-1_ and *bla*_PSZ-1_-like genes. The direction of genes is shown via an arrow. The *bla*_PSZ-1_ gene and putative *ampC* (*bla*_PSZ-1_-like) genes are colored red and the other genes are colored based on gene function classification. Those without direct names are depicted as *orfA*, YnfU family zinc-binding protein; *orfB*, MFS transporter; *orfC*, TetR/AcrR family transcriptional regulator; *orfD*, Hpt domain-containing protein; *orfE*, fimbrial protein; *orfF*, transporter substrate-binding domain-containing protein; *orfG*, molybdenum-dependent transcriptional regulator; *orfH*, pyridoxal phosphatase; *orfI*, DUF3289 family protein; *orfJ*, DUF943 family protein; *orfK*, kinase inhibitor; *orfL*, type 1 glutamine amidotransferase domain-containing protein; *orfM*, NADP-dependent oxidoreductase; and *orfN*, ATP-binding cassette domain-containing protein. The predicted hypothetical proteins (hp) are in dark gray. Regions with ≥80% amino acid identities are colored light gray.

The other two 20 kb sequences were from the genus *Erwinia*: *Erwinia rhapontici* BY21311 (NZ_CP085627) and *Erwinia rhapontici* MAFF 311155 (NZ_AP024333), and the upstream and downstream regions of the *bla*_PSZ-1_-like genes were almost completely different from those of *P. endophytica* X85, except for the *ampR*-*ampC* fragment. No mobile genetic element was identified in these 20 kb sequences. The upstream regions of all five sequences, including the one from the present study, had an *ampR* gene that encoded an AmpR protein (Figure 4). AmpR activates AmpC by binding anhydrous forms of cell wall precursor muropeptides, which are believed to act as cofactors for AmpC induction (Girlich et al., 2000). These findings suggested that the sequences from phylogenetically closer species had higher sequence identity and were more conserved in the related species.

## Conclusion

In this study, we described a novel AmpC β-lactamase gene, designated *bla*_PSZ-1,_ from an animal isolate *P. endophytica* X85, which shares the highest amino acid similarity (75.13%) with the function-characterized AmpC enzyme ERH-1; it shows resistance to penicillins and narrow-spectrum cephalosporins. The enzyme PSZ-1 also demonstrated hydrolytic activities against these antimicrobials, and the hydrolytic activity was strongly inhibited by the β-lactamase inhibitor avibactam. Research on the discovery of novel drug-resistance genes and their resistance mechanisms can help guide the scientific use of drugs in animal husbandry and animal/human clinical practice.

## Data availability statement

The datasets presented in this study can be found in online repositories. The names of the repository/repositories and accession number(s) can be found in the article/Supplementary material.

## Ethics statement

This study used strains obtained from an anal swab of a rabbit in an animal farm in Wenzhou, China. The owner of the farm was written informed of the study and expressed approval for sampling of animals. The studies involving human participants and animals were reviewed and approved by the Animal Welfare and Ethics Committee of Wenzhou Medical University, Zhejiang Province, China (Protocol number: wydw2021-0323).

## Author contributions

KL, HZ, QB, and LL: conceived and designed the experiments. JZ, YZ, YS, NL, GZ, JL, and TZ: performed the experiments. JZ, XZ, QL, and XL: data analysis and interpretation. JZ, QB, and LL: drafting of the manuscript. All authors contributed to the article and approved the submitted version.

## Funding

This study was supported by the Science & Technology Project of Wenzhou City, China (N20210001), the Science & Technology Project of Jinhua City, China (2022-2-013, 2022-4-017), and Zhejiang Provincial Natural Science Foundation of China (LY19C060002 and LQ17H190001).

## Conflict of interest

The authors declare that the research was conducted in the absence of any commercial or financial relationships that could be construed as a potential conflict of interest.

## Publisher’s note

All claims expressed in this article are solely those of the authors and do not necessarily represent those of their affiliated organizations, or those of the publisher, the editors and the reviewers. Any product that may be evaluated in this article, or claim that may be made by its manufacturer, is not guaranteed or endorsed by the publisher.

## Footnotes

^1^ https://tygs.dsmz.de/

^2^ http://www.bldb.eu/

^3^ https://www.expasy.org/resources/uniprotkb-swiss-prot/

^4^ https://itol.embl.de/

^5^ https://web.expasy.org/protparam/
